# Vitamin D Supplementation Improves Handgrip Strength in Postmenopausal Women: A Systematic Review and Meta-Analysis of Randomized Controlled Trials

**DOI:** 10.3389/fendo.2022.863448

**Published:** 2022-06-01

**Authors:** Jia-Li Zhang, Christina Chui-Wa Poon, Man-Sau Wong, Wen-Xiong Li, Yi-Xun Guo, Yan Zhang

**Affiliations:** ^1^ Spine Disease Research Institute, Longhua Hospital, Shanghai University of Traditional Chinese Medicine, Shanghai, China; ^2^ Key Laboratory of Theory and Therapy of Muscles and Bones, Ministry of Education, Shanghai, China; ^3^ Department of Applied Biology and Chemical Technology, The Hong Kong Polytechnic University, Hung Hom, Kowloon, Hong Kong SAR, China

**Keywords:** vitamin D, postmenopausal women, handgrip strength, muscle strength, mobility

## Abstract

**Introduction:**

In postmenopausal women, vitamin D deficiency (as defined by the circulating level of 25(OH)D being below 20 ng/ml (50 nmol/L)) is a regular occurrence. The effect of vitamin D supplementation on the muscle function of postmenopausal women has been controversial. This systematic review and meta-analysis of randomized controlled trials (RCTs) examines and summarizes the effects of vitamin D supplementation on the muscular strength and mobility of postmenopausal women.

**Methods:**

RCTs that met the inclusion criteria for this study were identified by searching PubMed, EMBASE, and the Cochrane Library. Postmenopausal women who were included in the study were exposed to RCTs assessing the effectiveness of vitamin D supplements. Meta-analysis data were extracted by two independent reviewers and screened for methodological quality. RCTs that did not meet the minimum requirement for assessment were excluded. In the meta-analysis, the effect size (weighted mean differences, WMD) of handgrip strength (HGS) and timed-up and go test (TUG) with a 95% confidence interval (CI) was obtained to compare reported results across the included RCTs.

**Results:**

A total of 19 trials were included in this systematic review, among which 13 trials were eligible for the meta-analysis. In the 13 included studies, supplementing with vitamin D produced a weighted mean difference of 0.876 kg (95% CI = 0.180 to 1.571, *P* = 0.014, I^2^ = 68.5%) for HGS, a measurement of muscle strength. However, an insignificant decrease of 0.044 s was observed after analyzing the TUG (95% CI = -0.979 to 0.892, *P* = 0.927, I^2^ = 95%). According to subgroup analysis, vitamin D supplementation increased HGS in patients over the age of 60 (*P* = 0.001), in those without calcium supplementation (*P* = 0.032), and in those whose baseline vitamin D level was greater than 75 nmol/L (30 ng/ml) (*P* = 0.003).

**Conclusions:**

Taking into account the studies in this systematic review, vitamin D supplementation improved muscle strength in postmenopausal women. However, an insignificant result was demonstrated in terms of mobility after vitamin D supplementation.

## Introduction

Postmenopausal women often have osteopenia and osteoporosis, mainly due to advanced age and lack of hormones, and this may cause a high incidence of falls and fractures (1–6). Several recent studies have suggested that muscle mass and bone mineral density (BMD) are linked among postmenopausal women (1–6). Furthermore, the loss of muscular mass and strength seen in postmenopausal women is the most basic symptom of sarcopenia, which is unanimously recognized by the academic consensus of experts from various continents (7–10).

Sarcopenia is caused by nutritional deficiencies, a sedentary lifestyle, decreased protein synthesis and regeneration, inflammation, hormonal and cytokine imbalances, and other factors (10, 11). Among these risk factors, vitamin D deficiency not only receives much attention in the academic medical community but also gradually gains higher awareness in diverse healthcare settings (12, 13).

Vitamin D deficiency is a common health problem among middle-aged people worldwide; it is estimated that more than 1 billion people are vitamin D deficient (6, 14–16). Epidemiological studies and investigations in various countries have revealed that vitamin D deficiency (serum 25(OH)D: less than 50 nmol/L) is common in postmenopausal women (14, 17, 18). Among them, 50.6% of Chinese postmenopausal women are vitamin D-insufficient, while 31.2% are vitamin D-deficient (19). According to compelling data, serum 25(OH)D levels are strongly linked to musculoskeletal function and muscular strength (20–23). Furthermore, multiple *in vivo* and *in vitro* experiments have indicated physiological and histological alterations associated with severe vitamin D deprivation, indicating that vitamin D supplementation has a favorable influence on musculoskeletal health. In detail, vitamin D supplementation is recommended by the Committee Recommendations for people at risk for vitamin D deficiency. They recommended that females who are over 51 years old need 1,500–2,000 IU, but less than 10,000 IU, of daily vitamin D intake (24). Additionally, the vitamin D receptor (VDR) has been identified in skeletal muscle tissue, muscle satellite cells, and myoblasts, which supports the idea that vitamin D might directly affect muscle tissue (25, 26).

International academic consensus emphasizes the importance of vitamin D supplementation or treatment for sarcopenia. Likewise, the impact of vitamin D on muscular function in various populations, namely, adults (27), older people (28–30), athletes (31–33), and patients with chronic diseases (34, 35) has been reported in systematic reviews and meta-analysis. There is, however, a lack of consistency in the results concerning the effect of vitamin D on muscle function in postmenopausal women based on several interventional studies. Vitamin D supplementation, alone or in conjunction with calcium supplements, has been shown in certain studies to enhance muscular strength and reduce the incidence of falls and fractures (36–39). However, several recent randomized controlled trials (RCT) on postmenopausal women with inadequate or deficient vitamin D levels found that vitamin D supplementation had no active effect on muscular strength or the number of falls (40, 41). As a result, there is still a pressing need for studies and academic consensus on postmenopausal women who are at high risk of vitamin D deficiency (7–9).

In this study, the data from the handgrip strength (HGS) and timed up and go test (TUG) are used to calculate the muscle function index, which is used to assess muscular strength and mobility, which are protracted indicators of fracture, osteoporosis, and sarcopenia in postmenopausal women. We conducted a meta-analysis on the efficacy of vitamin D supplementation on muscular strength and mobility in postmenopausal women based on a systematic evaluation of data from the chosen RCTs stated above.

## Methods

The current study was carried out in compliance with PRISMA (Preferred Reporting Items of Systematic Reviews and Meta-Analysis) recommendations (42).

### Literature Search

Two independent authors (J-LZ and W-XL) systematically searched PubMed, Embase, and the Cochrane Database from inception dates to January 2021 for randomized controlled trials that investigated the association between vitamin D supplementation and muscle atrophy using a predefined search algorithm using the following search algorithm: “((“muscular atroph*” OR “muscle atroph*” OR “Atroph* Muscular” OR “Atroph* Muscle” OR “sarcopenia*” OR “muscle strength” OR “mobility” OR “handgrip” OR “hand strength” OR “muscle function” OR “myatrophy” OR “myophagism” OR “physical performance”) AND (“postmenopausal women” OR “postmenopausal” OR “postmenopause” OR “PMP”)) AND (“vitamin d” OR “vitamin d” OR “25 hydroxyvitamin d” OR “vitamin d 2” OR “vitamin d 3” OR “calciferol*” OR “ergocalciferol*” OR “eldecalcitol*” OR “cholecalciferol*” OR “alphacalcidol*” OR “calcitriol*” OR “calcidiol*” OR “calcifediol*” OR “calciferol*” OR “dihydroxycholecalciferol”)”.

### Inclusion and Exclusion Criteria

After screening of titles and abstracts, duplicate studies and those clearly irrelevant were removed. The remaining full-texts of the papers were then retrieved to see if they matched the inclusion criteria for the current meta-analysis. The following are the inclusion criteria: 1) It had to be a human RCT with a cross-over or parallel design, 2) its study population had to be postmenopausal women, and 3) mean difference and standard deviations (SDs), standard error of the mean (SEMs), or 95% confidence intervals (CIs) were provided for the outcomes under consideration. The exclusion criteria are as follows: 1) *in vivo* animal studies, *in vitro* cell research, case reports, and observational studies; 2) any studies conducted in postmenopausal women with other diseases (such as type 2 diabetes, hypertension, trauma, etc.); 3) any studies investigating the effect of vitamin D supplementation combined with other interventions, such as nutrients like amino acid and protein supplements, physical exercise, other medication administration records like insulin, hormone therapy, and etidronate, etc.; 4) studies to be reported in other languages other than English; 5) RCTs that did not fulfill the minimal threshold for methodological quality evaluation.

### Data Extraction

The following data were extracted by two independent investigators (J-LZ and W-XL) from each included study: the first author, publication year; sample size (shown as: supplementation/control); age; supplementation; comparator; supplementation duration; presented data. Subgroup analysis was done on the basis of these pre-extracted data. Disagreements were resolved by consensus. Endnote X9 (Clarivate Analytics, Philadelphia, PA, USA) was used to undertake the extraction procedures.

### Assessment of the Quality of Included Literature

To estimate the quality of literature, the Jadad scale was used (43). This scale includes the following items: randomization, randomization scheme, double-blinding, double-blinding method, and dropouts and withdrawals in the supplementation and control groups. Each item would get a score from 0 to 1. In terms of quality, studies with scores of 0–2 are considered inferior, and those with scores of 3–5 are considered high quality (**Table 1**).

**Table 1:** Characteristics of included studies.

First author, year	Sample size (supplementation/control)	Age (years)	Supplementation	Comparator	Supplementation duration	Presented data	Jadadscore
**Apaydin, 2018 ** (44)	28/32	50–68	300,000 IU D_3_/single oral dose	800 IU D_3_/day	3 months	Knee extension; knee flexion	1
**Bischoff, 2003** (45)	62/60	63–99	800 IU D_3_/day	None	3 months	HGS; TUG; knee flexion; knee extension	5
**Bischoff-Ferrari, 2012** (46)	10/10	50–70	20 μg HyD/day; 140 μg HyD/week	800IU(D_3_)/per day; 5600IU(D_3_)/per week	4 months	TUG; knee flexion; knee extension; repeated sit-to-stand test;	4
**Bislev, 2018** (47)	40/41	60–80	2,800 IU D_3_/day	Placebo	3 months	HGS; TUG; elbow and knee extension flexion; chair raising test	4
**Brunner, 2008** (40)	1,185/1,162	50–79	400 IU D_3_/day	Placebo	60 months	HGS; chair stand test; time walk	4
**Cangussu, 2015** (36)	80/80	50–65	1,000 IU D_3_/day	Placebo	9 months	HGS; chair rising test	5
**Cheng, 2018** (37)	75/66	58.2 ± 8.1	0.5 μg calcitriol/day	Placebo	3 months	HGS	5
**Gao, 2015** (48)	101/109/251	63.44 ± 5.04	800 IU D_3_/day; 0.25 μg calcitriol/day	None	24 months	HGS; chair rising test	1
**Glendennig, 2012** (49)	353/333	>70	150,000 IU D_3_/3 months	Placebo	9 months	HGS; TUG	4
**Hansen, 2015** (50)	73/74/73	>60	800 IU D_3_/day; 50,000 IU D_3_/2 months	Placebo	12 months	TUG; five sit-to-stand test	5
**Hara,** **2013** (51)	50/44	55–75	1 μg alfacalcidol/day	Placebo	4 months	Back extensor strength	3
**Janssen,** **2010** (41)	36/34	>65	400 IU D_3_/day	Placebo	6 months	HGS; TUG; knee extension strength; leg extension power	5
**Meyer,** **2015** (52)	10/10	50–70	20 μg calcifediol/day	800 IU D_3_/day	4 months	Gait speed; trunk sway	5
**Mueangpaisarn, 2020** (53)	44/41	≥60	100,000 IU D_2_/week	40,000 IU D_2_/week	3 months	HGS; TUG	4
**Setiati,** **2018** (38)	46/42	≥60	0.5 μg alphacalcidol/day	Placebo	3 months	HGS; TUG	5
**Suebthawinkul, 2018** (54)	44/44	45–60	40,000 IU D_2_/week	Placebo	3 months	HGS; BIA; muscle cross-section area	5
**Uusi-Rasi,** **2015** (55)	88/96/95/91	70–80	800 IU D_3_/day; 800 IU D_3_/day +exercise	Placebo; Placebo +exercise	24 months	TUG; leg extensor strength; chair stand time; normal walking speed	5
**Verhaar,** **2000** (39)	10/14	≥70	0.25 μg alphacalcidol/day*1 month; 0.5 μg alphacalcidol/day*months	None	6 months	HGS; TUG; knee extension strength; walking test	1
**Zhu,** **2010** (56)	129/132	70–90	1,000 IU D_2_/day	Placebo	12 months	TUG; low limb muscle strength	5

HGS, handgrip strength; TUG, timed up and go test; HyD, 25-hydroxyvitamin D_3_; BIA, bioelectrical impedance analysis.

### Data Analysis

The effect sizes were expressed as weighted mean differences (WMDs), and the DerSimonian–Laird approach was used to calculate the 95% confidence interval (CI) from the random effects model. For each clinical study, WMDs were used to examine the effects of vitamin D supplementation on muscle outcomes such as HGS and TUG. The pooled effect sizes for each outcome were estimated using the altered value technique. We also used Cochrane’s Q test and I-square (I^2^) to assess any existing heterogeneity among the included RCTs. I^2^ greater than 50% with *P <*0.05 was applied to define heterogeneity (57). Furthermore, using the available moderator factors, sensitivity and subgroup analyses were performed to determine the impacts of each research paper on the pooled WMDs and to investigate the source of heterogeneity. Statistical analyses were carried out with STATA software version 15.0 (Stata Corp., College Station, TX) and Review Manager 5.4 software (Cochrane Collaboration, Oxford, UK).

## Results

### The Description of the Selected RCTs

**Figure 1:**
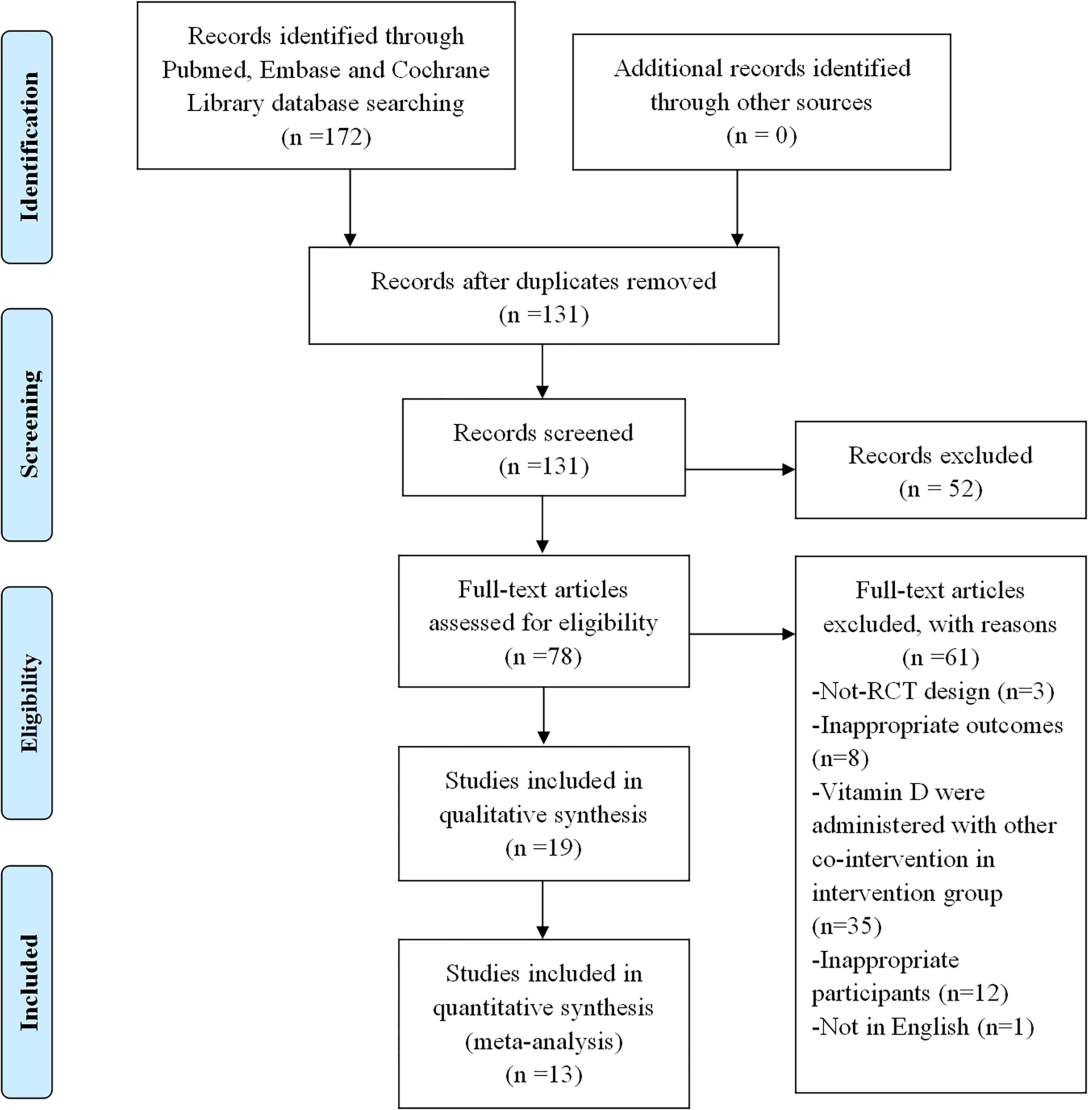
Flowchart of literature search for selection of trials. The selection process of the included trials has been shown in this figure. RCT, randomized controlled trial.

The first searches yielded a total of 172 potentially suitable documents, with 41 items being eliminated owing to the duplication. The titles and abstracts of the remaining 131 records were screened for inclusion. Then the full texts of 78 records were further estimated, and 19 met the inclusion criteria (36–41, 44–56), of which 13 of those 19 were suitable for meta-analysis. **Figure 1** shows a flowchart of the selection of RCT trials. The main characteristics of the RCTs are summarized in **Table 1**.

The year of publication of the 19 RCT trials included in this review was between 2003 and 2021, with 5,398 participants. The sample size of the RCTs included in multiple countries ranges from 20 (46, 52) to 2,347 (40). Furthermore, the duration of supplementation ranged from 3 (37, 38, 44, 45, 47, 53, 54) to 60 (40) months.

Different analogs of vitamin D_2_ (53, 54, 56) or vitamin D_3_ (36, 40, 41, 44–50, 52, 55), such as calcitriol (37, 48), alfacalcidol (38, 39, 51), and calcifediol (52), were used with different dosages in the RCT trials. In detail, vitamin D_3_ was applied in 12 of the 19 retrieved trials, whose dosages ranged from 400 IU/day (40, 41) to 300,000 IU in a single oral dose (44). Vitamin D_2_ was applied in 3 trials of the 19 selected studies at dosages ranging from 1,000 IU/day (56) to 100,000 IU/week (53), and calcitriol, alfacalcidol, and calcifediol were used in 6 trials of the 19 trials.

### Study Outcomes

In separate experiments, different approaches for measuring muscle function were used. Twelve (36–41, 45, 47, 49, 53, 48, 54) trials used HGS as a parameter indicating muscle strength. Other measurement methods included back extensor strength (51), low limb muscle strength (56), and leg extensor strength (55). The TUG was used to measure the mobility of subjects in 11 trials (38, 39, 41, 45–47, 49, 50, 53, 55, 56), while the timed walk (40), gait speed (52), walking test (39), etc., were used as the indicator of mobility in the remaining 8 trials.

The authors of 12 studies concluded that vitamin D supplementation with or without calcium played no significant role in the muscle strength and/or mobility of subjects, while 7 studies drew the opposite conclusion.

### Meta-Analysis

It was challenging to extract and analyze the data uniformly and compare the results since the data on muscular strength and mobility presented in the trials were acquired using a range of assessment methodologies. Additionally, HGS and TUG were the results of the more uniform and published data included in the studies, so we chose HGS as an index to measure the muscle strength of subjects (the best performance of HGS was obtained after three repeated measurements of the standing participants, holding the assessment for at least 5 s during the test with an interval of 30 s between each evaluation), and TUG as the indicator of the mobility measurement for this present meta-analysis.

### Handgrip Strength

**Figure 2:**
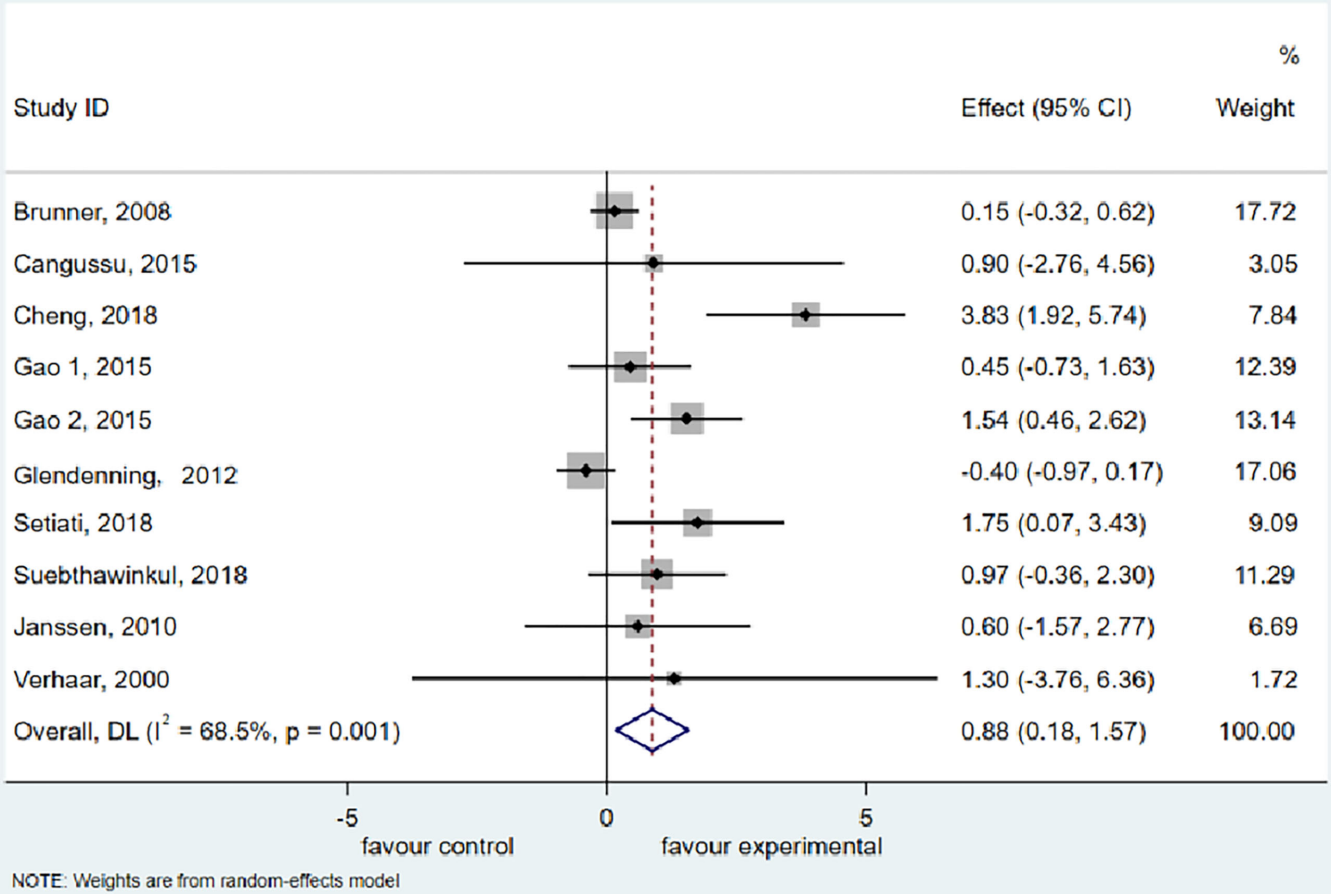
Forest plot displaying the effect of vitamin D supplementation on hand grip strength (HGS) using a random effects model. One study reported results as two intervention groups received two different dosages of vitamin D (Gao 1 and Gao 2). CI, confidence interval.

The meta-analysis of HGS included 9 trials (36–41, 48, 49, 54) with 1,997 participants supplemented with vitamin D and 2,232 participants as the control group (vitamin D in low dosage or placebo). Vitamin D supplementation resulted in considerable improvements in HGS (WMD: 0.876 kg, 95% CI = 0.180 to 1.571, *P* = 0.014), according to the analysis and finding over the random effects model. There was significant heterogeneity among studies (I^2^ = 68.5%, *P* = 0.001) (**Figure 2**).

### Timed Up and Go Test

**Figure 3:**
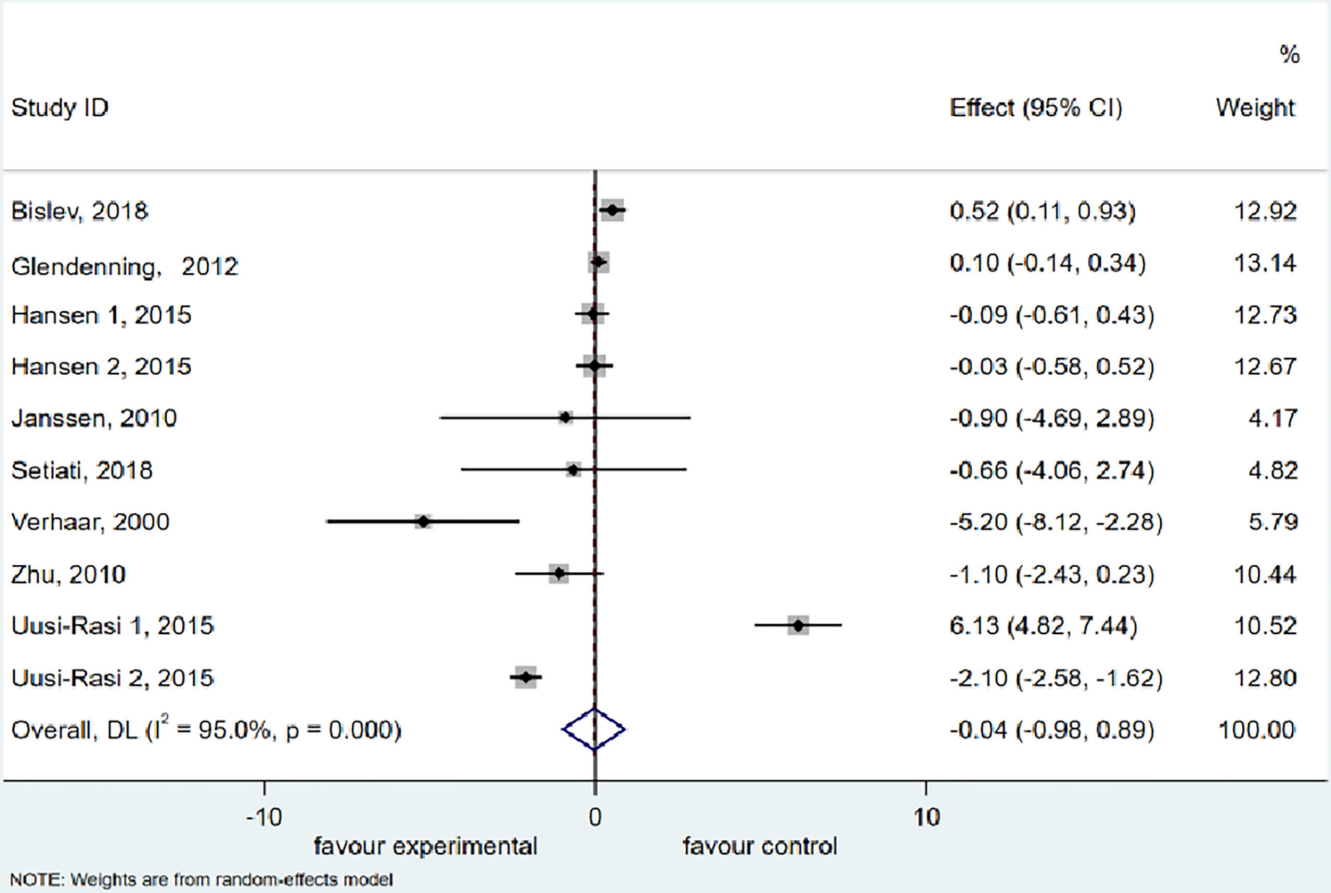
Forest plot displaying the effect of vitamin D supplementation on timed up and go test (TUG) using a random effects model. One study reported results as two intervention groups received two different dose of vitamin D (Hansen 1 and Hansen 2). CI, confidence interval.

The meta-analysis of TUG included 8 studies (38, 39, 41, 47, 49, 50, 55, 56) with 937 participants supplemented with vitamin D and 915 participants treated with placebo or a low dose of vitamin D. Using a random effects model, we discovered an insignificant decrease (0.044 s) in the TUG (95% CI = −0.979 to 0.892 s, *P* = 0.927) after vitamin D supplementation. Furthermore, the meta-analysis manifested a significant heterogeneity among the studies (I^2^ = 95.0%, *P* = 0.000) (**Figure 3**).

### Subgroup Analysis

We did the HGS and TUG subgroups analysis based on age, supplementation with calcium or not, vitamin D dosage (IU/day), supplementation duration (month), and baseline vitamin D level.

The scores of the heterogeneity of the HGS subgroups analysis were as follows: without calcium (I^2^ = 50.8%, *P* = 0.107); vitamin D dosage ≥1,000 IU/day (I^2^ = 47.0%, *P* = 0.152); supplementation duration <12 months (I^2^ = 74.0%, *P* = 0.001); baseline vitamin D <75 nmol/L (30 ng/ml) (I^2^ = 45.6%, *P* = 0.102) (**Table 2**).

**Table 2:** Subgroup analysis of HGS.

	Subtotal (n)	Number of studies (n)	WMD (95% CI)	*P*-value
**Age**				
>50	2,736	4	1.353 (−0.238; 2.944)	0.096
>60	870	4	1.116 (0.433; 1.799)	0.001
>70	623	2	−0.379 (−0.944; 0.186)	0.189
**With calcium or not**				
with calcium	3,816	6	0.478 (−0.165; 1.122)	0.145
without calcium	413	4	1.931 (0.166; 3.697)	0.032
**Baseline serum vitamin D level**				
<30 ng/ml	1,125	6	1.445 (0.486; 2.403)	0.527
>30 ng/ml	757	3	0.478 (−0.963; 1.918)	0.003
**Vitamin D dosage (IU/day)**				
<1,000	2,769	3	0.206 (−0.218; 0.630)	0.341
≥1,000	847	3	0.161 (−0.911; 1.234)	0.768
vitamin D analogs	613	4	2.118 (1.024; 3.213)	0
**Supplementation duration**				
>12	3,059	3	0.621 (−0.219; 1.461)	0.147
<12	1,170	7	1.193 (−0.090; 2.477)	0.068

WMD, weighted mean difference; CI, confidence interval.

Meanwhile, the scores of the heterogeneity of the TUG subgroups analysis were as follows: age >60 years old (I^2^ = 16.7%, *P* = 0.308); with calcium (I^2^ = 95.6%, *P* = 0.000); vitamin D dosage ≥1,000 IU/day (I^2^ = 57.8%, *P* = 0.069); supplementation duration <12 months (I^2^ = 76.1%, *P* = 0.002); baseline vitamin D >75 nmol/L (30 ng/ml) (I^2^ = 16.9%, *P* = 0.307) (**Supplementary Table**).

Moreover, according to subgroup analysis, vitamin D supplementation substantially raised HGS when compared to baseline blood vitamin D levels >75 nmol/L (30 ng/ml) (WMD = 0.478 kg, 95% CI = −0.963 to 1.918, *P* = 0.003), without calcium (WMD = 1.931 kg, 95% CI = 0.166 to 3.697, *P* = 0.032) and subject to an age of more than 60 (WMD = 1.116 kg, 95% CI = 0.433 to 1.799, *P* = 0.001).

### Publication Bias

Visual inspection of the funnel plot and Egger’s linear regression test revealed no indication of publication bias in the meta-analysis of vitamin D supplementation on HGS: *P* = 0.047 and TUG: *P* = 0.954.

## Discussion

This meta-analysis aimed to see if vitamin D supplementation might enhance the muscular strength and mobility of postmenopausal women. The results of the meta-analysis of the 13 trials included revealed a significant effect of vitamin D supplementation on the HGS of postmenopausal women, but not on the TUG. Since the HGS refers to muscle strength while the TUG represents locomotory function, it is considered that vitamin D supplementation could be effective in improving muscle strength in postmenopausal women. Thus, we assume that vitamin D supplementation might first enhance the upper extremities, but it is difficult to improve the strength of the lower limb muscles, which are more used in stance and mobility.

Since the data for men and women were not presented separately, trials that included both males and postmenopausal women were eliminated. Trials for postmenopausal women with diabetes (34), Parkinson’s disease (58), chronic kidney disease (35), or other special situations (59, 60) that might affect their mobility were also excluded. Moreover, some studies were considered inappropriate due to the use of combined vitamin D supplementation with other nutrients (61–65). All in all, 19 RCT trials were eventually included, and among them, 13 trials (36–41, 47–50, 54–56) were suitable for meta-analysis. Most of the studies were of medium to high methodological quality as assessed by the Jadad scale.

Nonetheless, because the studies examined varied in most dimensions, caution should be exercised in determining whether the characteristics of the researched people are consistent. Different techniques for measuring muscle function have been reported by different investigators, and even when measuring comparable parameters, separate methods have been used in different trials, making it even more difficult to compare results directly. For instance, muscle strength was measured in different ways, such as HGS (36–41, 45, 47–49, 53, 54), low limb muscle strength (56), and knee extension strength (39, 41). We used HGS to measure muscular strength, which has recently been proposed as a long-term predictor of fracture and mortality in postmenopausal women (66), and TUG is viewed as an outcome of mobility. Based on the findings of the included trials, taking vitamin D supplements significantly improved the strength of postmenopausal women. The primary outcomes of the quantitative meta-analysis suggested that vitamin D supplementation improved HGS (based on 9 studies) but played an insignificant role in decreasing the time of the TUG (based on 8 RCT studies). As a result, we concluded that vitamin D supplementation had a favorable effect on improving muscular strength in postmenopausal women.

However, the emerging reviews illustrate controversial conclusions about the impact of vitamin D supplementation on muscle function. Tabrizi et al. (67) and Abshirini et al. (68) demonstrated that taking vitamin D supplementation had no effect on markers of muscle function such as HGS and TUG in postmenopausal women, whereas some RCTs suggested a positive effect of vitamin D supplementation on muscle function in postmenopausal women (69–71). In this meta-analysis, however, we found a significant increase in HGS with a non-existent decrease in TUG in postmenopausal women after taking vitamin D supplementation. We assumed that the types of vitamin D, the duration of the supplementation, and the dose of vitamin D might account for the inconsistent conclusions as shown in **Table 1**. Additionally, given that the included RCTs displayed a high degree of heterogeneity, we conducted a subgroup analysis to reveal the potential source of heterogeneity. Following the subgroup analysis, we found that vitamin D supplementation enhanced HGS in participants aged no less than 60 years old, with a baseline level of vitamin D greater than 75 nmol/L (30 ng/ml) and without calcium supplementation during the period of the clinical trial. However, vitamin D supplementation was not found to have a significant effect on TUG from the results of any subgroup.

This study is the first meta-analysis to assess the effects of vitamin D supplementation on muscular health in healthy postmenopausal women enrolled in RCT studies. The strengths of the study are manifest in the fact that the populations studied were healthy postmenopausal women, and that the intervention strategy emphasized vitamin D supplementation or its analogs. However, there are also limitations in this meta-analysis, such as that the stringency of our exclusion criteria resulted in a lack of availability of physical activity, nutritional status, etc., which have the potentially contribute to the lack of change in TUG. Additionally, we also detected substantial heterogeneity between the included trials due to the large number of studies included and the diversity seen across different supplementation techniques. In this meta-analysis, we likewise identified no dose–response effect, which is likely due to the variety of supplementation programs used in the included studies. Furthermore, the limited database we searched was another flaw in this review.

The Asian working group for sarcopenia: 2019 consensus updates on sarcopenia diagnosis and treatment, which were published in JAMDA in 2020, suggested that interventions for patients with sarcopenia should include vitamin D-containing nutritional supplementation in addition to resistance exercise (7). In this review, we could only verify that supplementing with vitamin D is positively associated with muscle strength. This could be explained by the singleness of the supplementation, which we focused on in our review. The supplementation included in this meta-analysis not only excluded interventions such as branched-chain amino acids, whey protein, and other nutritional supplements, but also excluded treatment with resistance exercise, which is also considered an efficient means of intervention in the consensus.

Additionally, *in vitro* and *in vivo* experimental studies have demonstrated the beneficial effects of vitamin D supplementation on sarcopenia, which could be explained by the fact that vitamin D could bind to the VDR, which has been found in muscle tissue, myoblasts, and muscle satellite cells (25, 26). Moreover, the lack of vitamin D is suspected to cause muscle weakness by reducing the number and size of type II myofibers (72, 73), which are age-related fibers and the first to be recruited for balancing and preventing falls. Those facts might explain the association between circulating levels of 25(OH)D and muscle function in postmenopausal women (74). Thus, future studies that investigate the role of vitamin D supplementation on muscle should include an analysis of muscle composition. Furthermore, a recent study also reported an inverse relationship between skeletal muscle fat and circulating 25(OH)D levels in young women adults (75). Goodpaster et al. (76) and Liu et al. (77) have also found similar results that skeletal muscle attenuation is associated with the lipid content of skeletal muscle, which might thereby emphasize the significance of further research on skeletal muscle fat in postmenopausal women.

The findings of this systematic review and meta-analysis show that although vitamin D supplementation did not improve mobility, it did improve muscle strength, particularly in postmenopausal women over 60 years of age who are without calcium supplementation or whose baseline vitamin D is >75 nmol/L (30 ng/ml). These findings show that future trials should focus on determining the ideal dosage and duration and taking into account the several factors that may impair muscle performance, such as exercise, calcium consumption, frailty, a history of falls or fractures, and baseline vitamin D status, and the relationship between muscle function and/or strength with muscle composition.

## Data Availability Statement

The original contributions presented in the study are included in the article/**Supplementary Material**. Further inquiries can be directed to the corresponding author.

## Author Contributions

J-LZ designed and performed the research, analyzed data and wrote the paper. CP performed the research. M-SW designed and performed the research. W-XL performed the research, statistical analysis, and sample collection. Y-XG performed the research and sample collection. YZ designed and performed the research. All authors listed have made a substantial, direct, and intellectual contribution to the work and approved it for publication.

## Funding

This study was supported in part by the National Natural Science Foundation of China (82074468), the Science and Technology Commission of Shanghai Municipality (21400760400), the Program of Shanghai Academic Research Leader (19XD1423800), and the Shanghai Collaborative Innovation Center of Industrial Transformation of Hospital TCM Preparation.

## Conflict of Interest

The authors declare that the research was conducted in the absence of any commercial or financial relationships that could be construed as a potential conflict of interest.

## Publisher’s Note

All claims expressed in this article are solely those of the authors and do not necessarily represent those of their affiliated organizations, or those of the publisher, the editors and the reviewers. Any product that may be evaluated in this article, or claim that may be made by its manufacturer, is not guaranteed or endorsed by the publisher.
