# Supplementary material for: Vitamin D Supplementation Improves Handgrip Strength in Postmenopausal Women: A Systematic Review and Meta-Analysis of Randomized Controlled Trials
Source: Front Endocrinol (Lausanne). 2022 Jun 1;13:863448. doi: 10.3389/fendo.2022.863448 (PMC9199366; doi:10.3389/fendo.2022.863448)
Supplement: Supplementary file 1 [file DataSheet_1.doc]

**Supplement Table 1 Subgroup analysis of TUG**

|  | **Subtotal (n)** | **Number of studies (n)** | **WMD (95% CI)** | **p-value** |
| --- | --- | --- | --- | --- |
| **Age** |  |  |  |  |
| **>60** | 532 | 5 | 0.167(-0.161; 0.495) | 0.318 |
| **>70** | 1320 | 5 | -0.205(-2.223; 1.813) | 0.842 |
| **With calcium or not** |  |  |  |  |
| **with calcium** | 104 | 8 | 0.238(-0.895; 1.372) | 0.451 |
| **without calcium** | 1748 | 2 | -2.150(-7.743; 3.443) | 0.680 |
| **Baseline serum vitamin D level** |  |  |  |  |
| **<30 ng/ml** | 986 | 6 | -0.196(-1.947; 1.555) | 0.826 |
| **>30 ng/ml** | 866 | 4 | 0.232(-0.050; 0.513) | 0.106 |
| **Vitamin D dosage (IU/day)** |  |  |  |  |
| **<1000** | 625 | 4 | 0.839(-1.974; 3.653) | 0.559 |
| **≥1000** | 1116 | 4 | 0.117(-0.250; 0.485) | 0.532 |
| **vitamin D analogues** | 111 | 2 | -3.018(-7.646; 1.428) | 0.183 |
| **Supplementation duration** |  |  |  |  |
| **≥12** | 963 | 5 | 0.496(-1.334; 2.327) | 0.595 |
| **<12** | 889 | 5 | -0.138(-0.926; 0.650) | 0.731 |

WMD, weighed mean difference; CI, confidence interval.
